# Measuring sealant placement in children at the dental practice level

**DOI:** 10.1016/j.adaj.2020.06.015

**Published:** 2020-10

**Authors:** Shwetha V. Kumar, Alfa Yansane, Ana Neumann, Todd R. Johnson, Gregory W. Olson, Suhasini Bangar, Krishna Kumar Kookal, Aram Kim, Enihomo Obadan-Udoh, Elizabeth Mertz, Kristen Simmons, Joanna Mullins, Joel M. White, Elsbeth Kalenderian, Muhammad F. Walji

**Affiliations:** Department of Diagnostic and Biomedical Sciences, School of Dentistry, University of Texas Health Science Center at Houston, Houston, TX.; Department of Preventive and Restorative Dental Sciences, School of Dentistry, University of California, San Francisco, San Francisco, CA.; Department of General Practice and Dental Public Health, School of Dentistry, University of Texas Health Science Center at Houston, Houston, TX.; School of Biomedical Informatics, University of Texas Health Science Center at Houston, Houston, TX.; School of Dentistry, University of Texas Health Science Center at Houston, Houston, TX.; Department of Diagnostic and Biomedical Sciences, School of Dentistry, University of Texas Health Science Center at Houston, Houston, TX.; School of Dentistry, University of Texas Health Science Center at Houston, Houston, TX.; Department of Restorative Dentistry and Biomaterials Sciences, Harvard School of Dental Medicine, Harvard University, Cambridge, MA.; Department of Preventive and Restorative Dental Sciences, School of Dentistry, University of California, San Francisco, San Francisco, CA; Willamette Dental Group, Portland, OR.; Department of Preventive and Restorative Dental Sciences, School of Dentistry, University of California, San Francisco, San Francisco, CA.; lecturer, Harvard School of Dental Medicine, Harvard Univeristy, Cambrige, MA, and a professor, Department of Preventive and Restorative Dental Sciences, School of Dentistry, University of California, San Francisco, San Francisco, CA.; Department of Diagnostic and Biomedical Sciences, University of Texas School of Dentistry at Houston, 7500 Cambridge St, SOD 4184, Houston, TX 77054,

**Keywords:** Caries risk, dental quality measures, dental sealants, electronic measures

## Abstract

**Background.:**

Although sealants are an established and recommended caries-preventive treatment, many children still fail to receive them. In addition, research has shown that existing measures underestimate care by overlooking the sealable potential of teeth before evaluating care. To address this, the authors designed and evaluated 3 novel dental electronic health record–based clinical quality measures that evaluate sealant care only after assessing the sealable potential of teeth.

**Methods.:**

Measure I recorded the proportion of patients with sealable teeth who received sealants. Measure II recorded the proportion of patients who had at least 1 of their sealable teeth sealed. Measure III recorded the proportion of patients who received sealant on all of their sealable teeth.

**Results.:**

On average, 48.1% of 6- through 9-year-old children received 1 or more sealants compared with 32.4% of 10- through 14-year-olds (measure I). The average measure score decreased for patients who received sealants for at least 1 of their sealable teeth (measure II) (43.2% for 6- through 9-year-olds and 28.4% for 10- through 14-year-olds). Fewer children received sealants on all eligible teeth (measure III) (35.5% of 6- through 9-year-olds and 21% of 10- through 14-year-olds received sealant on all eligible teeth). Among the 48.5% who were at elevated caries risk, the sealant rates were higher across all 3 measures.

**Conclusions.:**

A valid and actionable practice-based sealant electronic measure that evaluates sealant treatment among the eligible population, both at the patient level and the tooth level, has been developed.

**Practical Implications.:**

The measure developed in this work provides practices with patient-centered and actionable sealant quality measures that aim to improve oral health outcomes.

Caries is one of the most preventable chronic diseases among school-aged children in the United States.^1^ Despite significant local and national efforts, it remains “one of the greatest unmet treatment needs among children.”^2^ The use of sealants has been proven effective in caries prevention,^3–11^ and increasing sealant use in children is perceived as a national health goal.^12^ Several programs have been launched toward achieving this goal, and consequently Healthy People 2020 progress reviews show an increase in sealant use from 25.5% through 28.1% for 6- through 9-year-olds and an increase from 19.9% through 21.9% for 13- through 15-year-olds.^13^

Health care quality is defined by the National Academy of Medicine as “the degree to which health services for individuals and populations increase the likelihood of desired health outcomes and are consistent with current professional knowledge.”^14^ Clinical quality measures are tools to help assess what level of health care quality we achieve. There is general agreement that to improve quality, measurement is the initial step. In our previous study, we evaluated sealant placement rates in dental practices, using specifications defined by the Dental Quality Alliance (DQA) and Oregon Health Authority (OHA), and we discovered that, at the practice level, both the OHA and DQA measures underestimated the care delivered. These dental quality measures are examples of process measures that report whether patients received recommended care. The DQA and OHA measures were originally designed for use by states and insurance plans that have access to claims data.^15^ When assessing the validity of these measures across 4 dental institutions, we identified measurement gaps that could be bridged by using data from the dental electronic health records (EHRs). We discovered that, at the practice level, both the OHA and DQA measures underestimated the care delivered. For example, approximately 67% of children who did not receive sealants had permanent first or second molars that were not sealable; that is, molars that had not erupted or were carious or had been previously sealed, restored, or extracted.^15^ The reason behind their inclusion could be that these metrics are restricted to using only the Current Dental Terminology (CDT) procedure codes documented in claims-based data to track care.^16^ The high-feasibility and low-cost implementation associated with using claims data is offset by its limited capture of clinical information and the lack of standardized and widely adopted diagnosis codes, making it difficult to precisely measure oral health outcomes and quality of life.^17^ Despite these concerns, federal and state programs and associations such as the Children’s Health Insurance Program,^18^ the California Dental Association, and Texas Medicaid have adopted these measures in their performance indicator tools.^19,20^

To overcome these limitations and develop a valid, reliable, and feasible quality metric, the use of EHR data is beneficial. As an organized, comprehensive repository, the vast and robust EHR facilitates the detailed data retrieval of clinical care and administrative records of all patients at the practice level,^21,22^ thus providing a more accurate and reliable assessment of a patient’s caries risk status. For example, through the EHR we are able to identify the number of caries-free molars eligible for sealants in children, an area in which claims data are limited.

The purpose of our study is to develop and validate a contemporary and usable electronic measure (e-measure), from EHR data, for all dental practices with EHR capabilities, to evaluate the proportion of children and adolescents who appropriately received a dental sealant on a permanent molar.

## METHODS

Our research group of public health care professionals, dentists, statisticians, and health care informaticians developed and tested the EHR-based sealant measure. The measure was implemented in 2 dental schools and 1 large dental accountable care organization. All 3 institutions belong to the Consortium for Oral Health Research and Informatics^23^ and use the axiUm EHR platform (Exan) and standardized diagnostic dental terminology (Systematized Nomenclature of Dentistry-Dental Diagnostic System [SNO-DDS]).^24^ Each participating institution obtained institutional review board approval before measure execution.

### Designing the e-measure

In a previous study, we assessed the performance of DQA and OHA claims–based sealant quality measures when adapted to the EHR and found gaps leading to underestimation of the sealant care provided.^15^ These gaps occurred because of the inherent gaps in the design of the claims-based measures to correctly characterize patients with teeth eligible for sealant treatment. For example, in the aforementioned study, in 43% of the patients who failed to meet the measure score numerator their first or second molars were unerupted. Therefore, we concluded that to estimate the true proportion of sealant placements within a given period, the measure first had to consider tooth eligibility before assessing sealant care. Using the American Dental Association’s evidence-based guidelines, a sealable tooth is a tooth likely to become carious (that is, deep pits and fissures) and have elevated risk.^25^ To accomplish this, we revised our electronic measures by first identifying patients with 1 or more “sealable teeth.” The term “sealable teeth” was introduced to define permanent molars eligible for sealant treatment, thereby excluding from our evaluation teeth that were unerupted, carious, restored, or previously sealed. In addition, we made a change to our numerator criteria by including the preventive resin restoration (PRR) procedure (CDT code D1352) in addition to the sealant procedure (CDT code D1351).^16^ PRR is similar to the sealant procedure, and both are treatments for enamel lesions. Using the same format detailed in our previous study, we evaluated our electronic measure by caries risk status, thereby running the measures both with and without the elevated caries risk constraint.

The measure relied on the following 4 risk assessment criteria within the EHR to identify the caries risk status of a pediatric patient:

- caries diagnosis with planned, in progress, or completed restorative, endodontic, fixed prosthetic, or surgical procedures;
- caries risk as documented in a caries risk assessment form;
- caries risk–diagnostic CDT codes;
- caries risk diagnosis and treatment pair, using a listing of caries risk diagnoses with concomitant preventive procedures.

#### Study Population

The inclusion criteria for our measure denominator included children aged 6 through 9 years and 10 through 14 years, who had completed either a periodic oral evaluation (CDT code D0120) or comprehensive oral evaluation (CDT code D0150) and who had at least 1 or more sealable teeth in a given reporting period.^16^ The age criterion for our study population was applied as of the last day of the reporting year. For the 6- through 9-year age group, only teeth nos. 3, 14, 19, and 30 were used to calculate the measure scores; for the 10- through 14-year age group, only teeth nos. 2, 15, 18, and 31 were used. To carry out the study objective, we designed 3 levels of quality measures to evaluate the sealant placement in an ordered form (Table 1):

- Level 1 (measure I score: sealant on any tooth). This measure recorded the proportion of patients who completed an evaluation (either CDT code D0120 or D0150) and who received a sealant (CDT code D1351) or a PRR (CDT code D1352) on a permanent first or second molar.
- Level 2 (measure II score: sealant on at least 1 sealable tooth). We restricted this measure to patients with eligible sealable teeth. Here the measure looked at the sealable-sealed combination across the 4 index teeth for both age groups. Patients with at least 1 sealable-sealed pairing were included in the numerator. This implied that only patients with at least 1 of their sealable teeth sealed were included in the measure.
- Level 3 (measure III score: sealants on all sealable teeth). This measure is similar to the measure II score but with more exacting criteria. In this case, only patients who had all of their sealable teeth sealed were included in the numerator. The purpose of this measure was to accurately estimate the proportion of patients who had all their sealable teeth sealed in the reporting year.

### EHR data extraction and data analysis

#### Step 1: Generating the Sampling Frame, Using the EHR

Because all the participating institutions were axiUm EHR users, we developed a Structured Query Language (SQL) script for all sites. The SQL queries generated a list of patients eligible to be included in both the denominator as well as the numerator. Each site tested the SQL query before the implementation.

#### Step 2: Measure Validation

To validate the query performance, we first ran the measure for a specific reporting period (2015) and age group (6–9 years) across the sites and compared performance with a manual review of charts.

#### Step 2a: Sample size estimation

The number of charts needed for manual review was calculated, using the sample size formula for estimating a proportion. The sample size calculation used the following initial values: the measure score proportion estimate, the standard margin for error (0.05), and the 2-tailed *z* score (*z*, 1.96) value.

#### Step 2b: Validation of the automated query

Similar to our previous studies,^15,26–29^ we trained and calibrated 2 reviewers from each site to audit sampled charts. The first 50 charts from a randomized list of patients were analyzed by each of the reviewers for interrater reliability to establish calibration. Only when the 2 reviewers achieved a *κ* correlation coefficient greater than 0.80 (*κ* = 0.80) were they allowed to individually review the remaining charts.^30^ Sensitivity, specificity, positive predictive value, and negative predictive value of the automated query were calculated, using the manual review as the reference standard. Once validated, we executed the measures across 4 consecutive years at all sites.

### Statistical analysis

Descriptive statistics were calculated for demographic variables as well as each measure score, and bar charts were used for graphical representation. To determine whether there were significant age variations in the measure scores, an independent sample *z* test was performed. To determine whether elevated caries risk was associated with increases in the measure scores, a *z* test for proportions was completed. All tests were conducted at the standard significance level (.05), and the Benjamini-Hochberg false discovery rate method was used to adjust all *P* values. All analyses were performed with statistical software (Stata, StataCorp) and R (R Project for Statistical Computing).^31^

## RESULTS

Our query retrieved 181,565 patients from the 3 participating organizations. The demographic characteristics of our study population are listed in Table 1. The most frequently reported age within our sample population was 10 years with a nearly equal distribution of male and female patients (male patients, 50.0%; female patients, 49.9%; unknown, 0.1%). Of our study population, 47.4% identified as white, 6.2% as Asian, 5.0% as black, 18.8% as Hispanic, 1.0% as Native American, 0.9% as Native Hawaiian or Pacific Islander, and 20.7% unknown. Notwithstanding 21% of the population with unknown race information, the distribution remained consistent across the various age groups. Our caries risk assessment criteria deemed nearly one-half of our population to be at an elevated caries risk (48.5%).

The validity for each sealant measure was established using sensitivity, specificity, positive predictive value, negative predictive value, and the Cohen *κ* correlation coefficient to compare the results found in the manual reviews to the automated query. The *κ* correlation coefficient compared individual reviewer scores for a measure with the electronically generated scores for the same measure. Among all the sites, there was an average *κ* of 86.3% (*κ* = 86.3%), which represents an “almost perfect agreement.”^30^ For the sealant measure without elevated caries risk, the site 2 *κ* value (*κ* = 38.2%) and positive and negative predictive values were found to be lower than for other sites. We deduced the reason for this low validity to be an error in the script, leading to incorrect inclusion of patients with unerupted molars. We rectified this error in our elevated caries risk iteration by updating the script.

**Figure 1.**
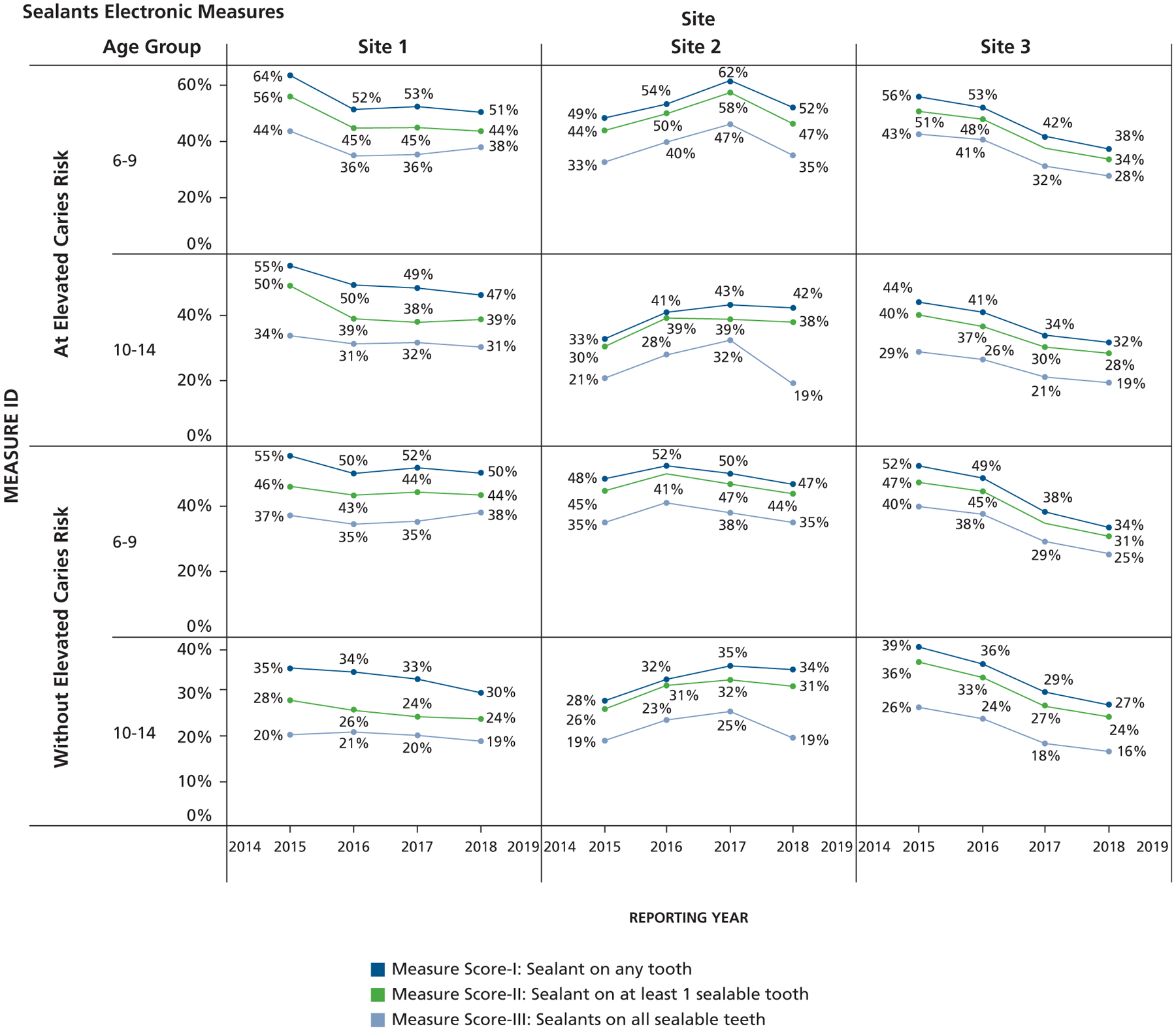
Overall scores for years 2015 through 2018 for sealant measures in patients with and without elevated caries risk.

**Figure 2.**
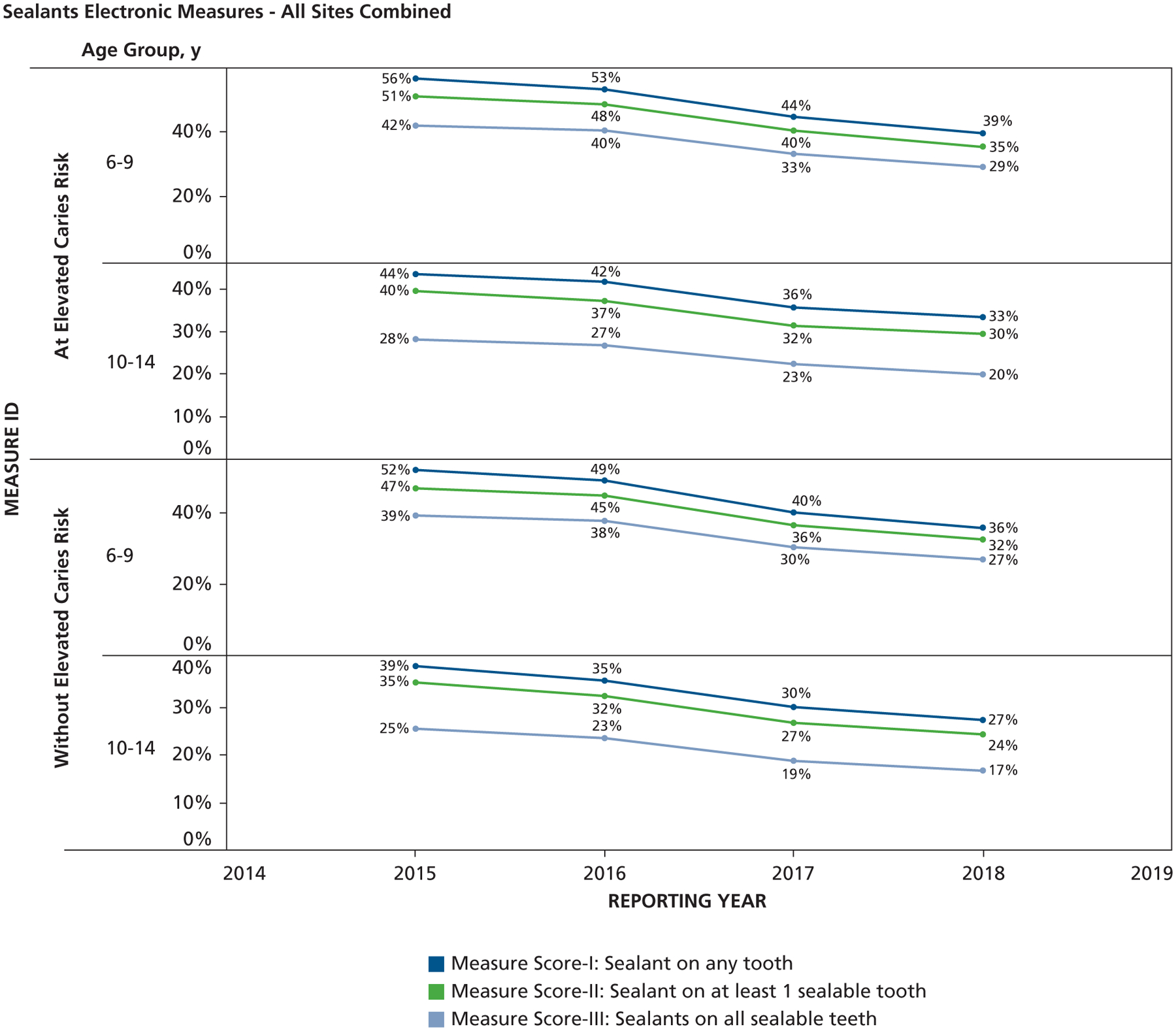
Scores for years 2015 through 2018 for sealant measures in patients with and without elevated caries risk across 3 sites.

Overall, as shown in Figure 1, while leaving out caries risk, the scores were highest for measure I, followed by measure II, and measure III. The average measure scores for all sites combined and stratified by age categories were 48.1%, 43.2%, and 35.5% in 6- to 9-year-olds and 32.4%, 28.4%, and 21% in 10- to 14-year-olds for measures I, II, and III, respectively. Among patients at elevated caries risk, who accounted for 48.5% of the total study population, the scores for measures I, II, and III were all higher than those assessed irrespective of caries risk. The measure scores varied by site and also over a 4-year period. Figure 2 shows the combined 4-year data, in which we observed that the sealant measure scores were higher for patients with elevated caries risk across sites. There were also significant differences in all measure scores across age groups for all sites. Table 2 shows the results of the independent sample hypothesis test comparing the differences in measure scores by age for patients with and without elevated caries risk. We see that irrespective of caries risk status, there were significant differences in measure performance across age groups. The measure scores for the 6- through 9-year age group were higher than those for the 10- through 14-year age group. Table 3 shows the results of the independent sample hypothesis tests comparing the statistical differences in measure scores by elevated caries risk status. At site 1, there were no significant differences in all measure scores by caries risk status for the 6- through 9-year-old age group. At site 2, there were significant differences in measure scores I and II by caries risk status for the 6- through 9-year-old age group, but no differences were found for measure III. For site 3, there were significant differences in all measure scores by caries risk status for the 6- through 9-year-old age category. For the 10- through 14-year age group, there were significantly higher measure scores for patients with elevated caries risk than those without risk.

## DISCUSSION

In our prior work, we showed the shortcomings of claims-based sealant measures in identifying patients with eligible teeth for sealant treatment, thus underestimating the care delivered.^15^ We have attempted to overcome this limitation by developing an algorithm that uses EHR data to include patients with 1 or more sealable teeth and by excluding patients with restored, previously sealed, carious, or missing permanent first or second molars. To arrive at a comprehensive preventive process of care performance, we included both pit-and-fissure sealants as well as PRR as our numerator criteria. We also developed 3 perspectives by which to assess the performance of a dental practice. First, we report the percentage of children who received a sealant or PRR on any tooth. On testing, we encountered patients without sealable teeth receiving sealant retreatment. To account for these oddities, the second measure was designed to determine the percentage of children who received a sealant or PRR on 1 or more sealable teeth. We found minor differences in scores for these 2 measures. Last, the third measure determined the percentage of children who received a sealant on all of their sealable teeth. We found that practices performed lower with more stringent criteria when measuring if all sealable teeth received a sealant.

There is substantial evidence on the effectiveness of sealants in preventing caries and helping with arresting pit-and-fissure caries.^6^ A 2016 systematic review reported that children and adolescents who received sealants on noncavitated occlusal surfaces experienced fewer new carious lesions as compared with those without sealants.^32^ Evidence-based clinical recommendations for the use of pit-and-fissure sealants, released by the American Dental Association in 2008, were designed to aid practitioners in the development of a relevant sealant treatment plan.^3^ However, despite these guidelines, studies have found that established clinical practice attitudes hamper the translation of these guidelines to everyday practice.^33–35^ Our results show that barriers exist in following evidence-based guidelines, and that they need to be identified and eliminated to allow for more timely execution of preventive dental care.

Although there were differences in measure scores across the 3 participating institutions, we did find higher scores for those with elevated caries risk. Caries risk is based on the balance of disease indicators, risk factors, and protective factors. Incipient initial enamel lesions in pits and fissures and smooth surfaces are disease indicators that elevate caries risk to “moderate caries risk.” Frank caries into dentin is also a disease indicator that elevates caries risk to “high caries risk.” Throughout this study, guidelines dictated that the practitioner perform a caries risk assessment to determine whether a sealant procedure was indicated, but revised guidelines from 2020 have removed this stipulation. Some may argue that sealing teeth in all children is better than selectively having to decide whether the patient is at high caries risk. We agree that for large-scale, public health care efforts, when the dental team can treat a child only once, sealing all teeth in all children may be appropriate. However, for dental practices in which parents or guardians may bring their children in routinely for regular pediatric oral care, targeted sealants to those who are at elevated risk may be more prudent. First, it frees up the dental team and dental operatory for those patients who need care; second, it lowers cost for the patient or the insurer; third, it prevents potential sequelae later when the sealant becomes partially or fully lost.^25^ Other reasons for variability in sealant placement may be due to educational pressures (in academic sites), treatment planning philosophies, and the institutional culture and environment. Sealants are indicated for retentive pits and fissures likely to become carious. Caries risk is a factor in the determination of whether a retentive pit and fissure is likely to become carious and whether a sealant is indicated. We therefore developed these measures stratified by caries risk.

The results of our study bolster the usefulness of the EHR while supporting accurate measurement of dental quality.^26–29^ In 2012, the DQA similarly proposed an e-measure for sealants for 6- through 9-year-old children.^36^ We hope that with the increased adoption of EHRs in practice and the collection of key data such as structured diagnoses,^24^ dental clinics will be prompted to systematically measure the quality of care provided. Although our study was conducted in institutions that use the same EHR, the measures can be implemented in other EHRs. We envision these types of practice-level quality measures aiding everyday providers to improve quality.^37^ It would allow for regular measuring, adjusting, and remeasuring of any given area for improvement. We recognize that the use of HER-based measures may pose a cost for some practices and that dentistry must strike a balance between the accuracy and usefulness of its measures. This flexibility in measurement will allow for a wider range of dental practices to evaluate their sealant placement rates, but additional study is required to fully understand the costs and benefits of EHR measure use. We are also aware that there is a separate debate on how quality measures should be used, and if they should be tied to reimbursements or incentives.^38^ There may be other unintended consequences after these measures are implemented in practice that need further study.

Our e-measure queried structured data and was able to capture existing and past dental experiences by harnessing the ability of EHR databases to record real-time care. We were also able to assess sealant placement comprehensively by measuring 3 ways to recognize the level of care. We again showed that data from EHRs can be used to accurately assess whether children received sealants, and, if so, on most or all eligible teeth.

## LIMITATIONS

We recognize that the proposed measures cannot be used in clinics or other sealant-based programs that do not use an EHR. Our measure was also developed in the axiUm EHR, and we do not have experience yet in implementing the measure in other EHRs. Our e-measure operated on the assumption that the standards of data documentation were also upheld. This measure was also designed to be reported annually. Therefore, we did not adjust the measure numerator on the basis of a qualifying examination date. So, it is possible that children who were seen later in the reporting year would have had less time to be included in the numerator than those seen earlier in the same reporting year. In addition, our study design looked at cross-sectional data rather than longitudinal data to measure sealant placement. It would be informative to observe how each patient from the ages of 6 through 14 differs in caries risk, sealant eligibility, and other associated factors influencing preventive care by using longitudinal data spanning multiple years. Last, this measure does not consider substandard recall, excludes restored teeth, and includes only unrestored teeth that have been adequately sealed. But these limitations apply to all measures that exclude teeth that have been restored or are carious, and this measure was not designed to show substandard recall care.

## CONCLUSIONS

We developed and validated a practice-based sealant e-measure that assesses sealant treatment in the eligible population, both at the patient level and the tooth level. Although the purpose of this study was to develop and assess the e-measure, the results also show that sealant use among the 3 sites in this study was lower than desired given the overwhelming evidence on the benefits of sealants for preventing caries, implying that further quality improvement is needed.

## Figures and Tables

**Table 1.** Demographic characteristics of the study population.

CHARACTERISTIC	AGE, y									
	6	7	8	9	10	11	12	13	14	TOTAL
**Participants, No.**	18,613	19,503	20,228	20,485	20,751	20,758	20,550	20,555	20,122	181,565
**Sex, No. (%)**										
Female	9,255 (49.7)	9,702 (49.8)	10,058 (49.7)	10,133 (49.5)	10,337 (49.8)	10,308 (49.7)	10,241 (49.8)	10,392 (50.6)	10,122 (50.30)	90,548 (49.9)
Male	9,351 (50.2)	9,796 (50.2)	10,163 (50.2)	10,346 (50.5)	10,392 (50.1)	10,429 (50.2)	10,274 (50.00)	10,130 (49.3)	9,956 (49.5)	90,837 (50.0)
Unknown	7 (0.04)	5 (0.03)	7 (0.03)	6 (0.03)	22 (0.1)	21 (0.10)	35 (0.2)	33 (0.2)	44 (0.2)	180 (0.10)
**Race, No. (%)**										
White	8,269 (44.4)	8,816 (45.2)	9,270 (45.8)	9,537 (46.6)	9,759 (47.0)	9,995 (48.2)	10,038 (48.9)	10,169 (49.5)	10,112 (50.3)	85,965 (47.4)
Asian	1,372 (7.4)	1,352 (6.9)	1,342 (6.6)	1,306 (6.4)	1,270 (6.1)	1,249 (6.0)	1,175 (5.7)	1,140 (5.6)	1,100 (5.5)	11,306 (6.2)
Black	1,074 (5.8)	1,026 (5.3)	1,090 (5.4)	1,072 (5.2)	1,042 (5.0)	954 (4.6)	977 (4.8)	930 (4.5)	890 (4.4)	9,055 (5.0)
Hispanic	3,837 (20.6)	4,017 (20.6)	3,944 (19.5)	4,036 (19.7)	3,973 (19.2)	3,745 (18.0)	3,612 (17.6)	3,642 (17.7)	3,332 (16.6)	34,138 (18.8)
Native American	165 (0.9)	176 (0.9)	200 (1.0)	196 (0.9)	225 (1.1)	222 (1.1)	235 (1.1)	202 (0.9)	210 (1.0)	1,831 (1.0)
Native Hawaiian or Pacific Islander	176 (0.9)	193 (1.0)	211 (1.0)	194 (0.9)	205 (1.0)	194 (0.9)	179 (0.9)	178 (0.9)	192 (0.9)	1,722 (0.9)
Unknown	3,720 (20.0)	3,923 (20.1)	4,171 (20.6)	4,144 (20.2)	4,277 (20.6)	4,399 (21.2)	4,334 (21.1)	4,294 (20.9)	4,286 (21.30)	37,548 (20.7)
**Elevated Caries Risk, No. (%)**	9,603 (51.6)	10,728 (55.0)	11,428 (56.5)	11,252 (54.9)	10,245 (49.4)	9,235 (44.5)	8,391 (40.8)	8,477 (41.2)	8,690 (43.2)	88,049 (48.5)

**Table 2.** Differences in measure scores across age.

			6 TO 9 YEARS OF AGE		10 TO 14 YEARS OF AGE				
CARIES RISK STATUS	SITE	MEASURE DESCRIPTION	%	95% CI^†^	%	95% CI	DIFFERENCE (%)	*z* TEST	*P* VALUE*
**At Elevated Caries Risk**	1	Measure I score	54.2	0.52 to 0.56	49.6	0.47 to 0.52	4.5	2.74	.0073
		Measure II score	47.0	0.45 to 0.49	40.8	0.38 to 0.43	6.2	3.55	.0005
		Measure III score	37.9	0.36 to 0.40	31.7	0.29 to 0.34	6.3	3.71	.0003
	2	Measure I score	53.7	0.52 to 0.56	39.5	0.37 to 0.42	14.3	8.46	< .0001
		Measure II score	49.6	0.47 to 0.52	36.3	0.34 to 0.39	13.3	7.71	< .0001
		Measure III score	38.8	0.37 to 0.41	25.0	0.23 to 0.27	13.7	8.41	< .0001
	3	Measure I score	46.5	0.46 to 0.47	37.6	0.37 to 0.38	8.8	17.48	< .0001
		Measure II score	42.1	0.41 to 0.43	33.5	0.33 to 0.34	8.7	17.02	< .0001
		Measure III score	35.2	0.35 to 0.36	23.4	0.23 to 0.24	11.8	24.48	< .0001
**At Low Caries Risk**	1	Measure I score	51.8	0.50 to 0.54	32.0	0.30 to 0.34	19.9	15.00	< .0001
		Measure II score	44.3	0.42 to 0.46	25.5	0.24 to 0.27	18.8	13.81	< .0001
		Measure III score	36.2	0.34 to 0.38	20.0	0.18 to 0.22	16.2	12.62	< .0001
	2	Measure I score	49.4	.48 to 0.51	32.6	0.31 to 0.34	16.7	13.52	<.0001
		Measure II score	46.2	0.45 to 0.48	30.1	0.28 to 0.32	16.2	12.94	< .0001
		Measure III score	37.5	0.36 to 0.39	22.0	0.20 to 0.24	15.5	13.04	< .0001
	3	Measure I score	43.0	0.43 to 0.44	32.7	0.32 to 0.33	10.3	30.53	< .0001
		Measure II score	39.1	0.39 to 0.40	29.7	0.29 to 0.30	9.3	27.56	< .0001
		Measure III score	32.8	0.32 to 0.33	21.0	0.20 to 0.21	11.8	37.41	< .0001

* All *P* values are adjusted, using the Benjamini-Hochberg false discovery rate method.

† CI: Confidence interval.

**Table 3.** Comparison of sealant measure scores by caries risk status.

AGE, Y	SITE	MEASURE DESCRIPTION	ALL CARIES RISK		ELEVATED CARIES RISK		DIFFERENCE (%)	Z TEST	P VALUE*
			%	95% CI^†^	%	95% CI			
**6–9 y**	1	Measure I score	51.8	0.50 to 0.54	54.2	0.52 to 0.56	2.3	−1.68	.0988
		Measure II score	44.3	0.42 to 0.46	47.0	0.45 to 0.49	2.7	−1.81	.0764
		Measure III score	36.2	0.34 to 0.38	37.9	0.36 to 0.40	1.8	−1.24	.2219
	2	Measure I score	49.4	0.48 to 0.51	53.7	0.52 to 0.56	4.4	−3.36	.0009
		Measure II score	46.2	0.45 to 0.48	49.6	0.47 to 0.52	3.3	−2.48	.0152
		Measure III score	37.5	0.36 to 0.39	38.8	0.37 to 0.41	1.3	−0.99	.3225
	3	Measure I score	43.0	0.43 to 0.44	46.5	0.46 to 0.47	3.4	−8.33	< .0001
		Measure II score	39.1	0.39 to 0.40	42.1	0.41 to 0.43	3.1	−7.32	< .0001
		Measure III score	32.8	0.32 to 0.33	35.2	0.35 to 0.36	2.4	−6.31	< .0001
**10–14 y**	1	Measure I score	32.0	0.30 to 0.34	49.6	0.47 to 0.52	17.7	−11.24	< .0001
		Measure II score	25.5	0.24 to 0.27	40.8	0.38 to 0.43	15.3	−9.59	< .0001
		Measure III score	20.0	0.18 to 0.22	31.7	0.29 to 0.34	11.7	−7.95	< .0001
	2	Measure I score	32.6	0.31 to 0.34	39.5	0.37 to 0.42	6.9	−4.37	< .0001
		Measure II score	30.1	0.28 to 0.32	36.3	0.34 to 0.39	6.2	−3.95	.0001
		Measure III score	22.0	0.20 to 0.24	25.0	0.23 to 0.27	3.0	−2.15	.0355
	3	Measure I score	32.7	0.32 to 0.33	37.6	0.37 to 0.38	4.9	−11.20	< .0001
		Measure II score	29.7	0.29 to 0.30	33.5	0.33 to 0.34	3.7	−8.62	< .0001
		Measure III score	21.0	0.21 to 0.21	23.4	0.23 to 0.24	2.4	−6.29	< .0001

* All *P* values are adjusted, using the Benjamini-Hochberg false discovery rate method.

† CI: Confidence interval.

## ABBREVIATION KEY

CDT: Current Dental Terminology
DQA: Dental Quality Alliance
EHR: Electronic health record
E-measure: Electronic measure
OHA: Oregon Health Authority
PRR: Preventive resin restoration
SQL: Structured Query Language
